# A Candidate Approach Implicates the Secreted *Salmonella* Effector Protein SpvB in P-Body Disassembly

**DOI:** 10.1371/journal.pone.0017296

**Published:** 2011-03-01

**Authors:** Ana Eulalio, Kathrin S. Fröhlich, Miguel Mano, Mauro Giacca, Jörg Vogel

**Affiliations:** 1 RNA Biology Group, Max Planck Institute for Infection Biology, Berlin, Germany; 2 Institute of Molecular Infection Biology, Würzburg University, Würzburg, Germany; 3 Molecular Medicine Laboratory, International Centre for Genetic Engineering and Biotechnology, Trieste, Italy; University of Birmingham, United Kingdom

## Abstract

P-bodies are dynamic aggregates of RNA and proteins involved in several post-transcriptional regulation processes. P-bodies have been shown to play important roles in regulating viral infection, whereas their interplay with bacterial pathogens, specifically intracellular bacteria that extensively manipulate host cell pathways, remains unknown. Here, we report that *Salmonella* infection induces P-body disassembly in a cell type-specific manner, and independently of previously characterized pathways such as inhibition of host cell RNA synthesis or microRNA-mediated gene silencing. We show that the *Salmonella*-induced P-body disassembly depends on the activation of the SPI-2 encoded type 3 secretion system, and that the secreted effector protein SpvB plays a major role in this process. P-body disruption is also induced by the related pathogen, *Shigella flexneri*, arguing that this might be a new mechanism by which intracellular bacterial pathogens subvert host cell function.

## Introduction

A significant fraction of cellular messenger RNAs are not translated immediately after their export from the nucleus to the cytoplasm, being subjected to quality control and regulatory mechanisms that lead to mRNA degradation or translation repression. These different fates of transcripts have been associated with specific subcellular localizations: mRNAs being actively translated are associated with polysomes; mRNAs targeted for degradation or translation repression accumulate in P-bodies (a.k.a. mRNA processing bodies or GW bodies); and, in response to stress conditions, mRNAs stalled in translation initiation accumulate in stress granules.

P-bodies (henceforth, PBs) are dynamic cytoplasmic structures containing small regulatory and messenger RNAs, as well as proteins associated with post-transcriptional regulations such as mRNA decay, translational repression, mRNA surveillance and RNA-mediated gene silencing [1], [2]. PB formation is at least partially, a consequence of RNA silencing and decay, though not necessarily essential for these pathways [3], [4], [5], [6]. Stress granules (SGs) are a different type of cytoplasmic structures, composed of dynamic aggregates of RNA binding proteins, a subset of translation initiation factors, 40S ribosomal subunit and mRNAs. In contrast to the constitutive presence of PBs, SG form upon different environmental stresses that inhibit translation initiation, for instance, heat-shock, oxidative stress, UV irradiation or energy starvation [1], [7], [8]. PBs and SGs are often detected docked to each other, and it has been suggested that mRNAs can move between these two compartments [9].

There has been accumulating evidence to implicate PBs, SGs, and their protein components in the regulation of RNA metabolism during viral infections, with clear consequences for the life cycle of various viruses [10]. For example, PB components were found to be required for Brome mosaic virus and Hepatitis C virus replication [11], [12], [13], [14], whereas disruption of PBs was shown to enhance HIV-1 production and infectivity [15]. Recently, poliovirus infection was shown to induce PB disruption during the mid-phase of the infection cycle [16]. Additionally, West Nile and Dengue viruses hamper PB assembly later during infection, although both the mechanism and outcome for infection are yet unknown [17]. Viral infection has also been shown to affect the assembly of SGs, either positively by polioviruses and reoviruses [18], [19], or negatively by rotavirus and flaviviruses [17], [20]. A repression of SG formation might facilitate the translation of viral mRNAs; some SG components can limit viral infection, suggesting that formation of SGs might be part of the host antiviral response [10].

Similar to viruses, intracellular bacterial pathogens extensively manipulate host cellular pathways to ensure their survival and replication, impacting on host cell cytoskeleton, signal transduction pathways, membrane trafficking and pro-inflammatory responses, to name a few [21], [22], [23]. Unlike for viral pathogens, however, effects of bacterial pathogens on PBs and SGs have not been addressed. In this paper we investigate this matter with the bacterium *Salmonella enterica* serovar Typhimurium (hereafter, *Salmonella*), a Gram-negative model pathogen that targets a variety of eukaryotic hosts to cause different diseases ranging from gastroenteritis to typhoid fever [24], [25]. *Salmonella* can infect nonphagocytic cells, in particular epithelial cells, by actively promoting its own internalization and its intracellular survival. These processes are primarily mediated by two type 3 secretion systems (T3SS) that are encoded on the *Salmonella* pathogenicity island 1 and 2 (SPI-1 and SPI-2). The T3SS inject bacterial effector proteins directly into the host cell cytoplasm, to facilitate bacterial invasion (SPI-1), and intracellular replication (SPI-2) within the *Salmonella*-containing vacuole (SCV) [21], [22], [23].

We report here that *Salmonella* infection induces a novel type of PB disassembly in epithelial cells that is unrelated to previously determined mechanisms such as the inhibition of host cell transcription or microRNA-mediated gene silencing. Using a collection of bacterial effector mutants, we determined that PB disassembly is dependent on the activity of the SPI-2 encoded T3SS, and identified the secreted effector protein, SpvB, as a mediator of this process. Finally, we demonstrate that the effect of bacterial infection on PB integrity is not limited to *Salmonella*, and that *Shigella flexneri* also induces PB disassembly.

## Results

### *Salmonella* infection induces PB disassembly

**Figure 1:**
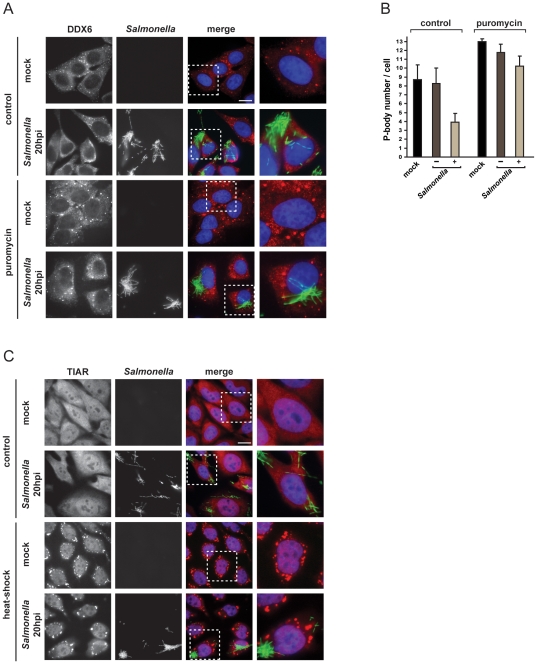
*Salmonella* infection induces PB disassembly, but not SG formation. (A) HeLa cells were mock-treated or infected for 20 hours with *Salmonella* expressing GFP, and either left untreated (control) or treated with puromycin for 1 hour. PBs were detected by staining the cells with anti-DDX6 antibody (red channel). Scale bar, 10 µm. The region highlighted by a white square is enlarged on the rightmost panel. (B) The average number of PBs per cell in mock-treated cells and *Salmonella* infected cells was calculated as described in Materials and Methods. *Salmonella* infected cells were divided into two populations: cells in which the bacteria were not internalized (*Salmonella* −) and cells in which the bacteria were internalized (*Salmonella* +). Mean values ± standard deviations from at least three independent experiments are shown. (C) SG formation was evaluated in HeLa cells mock-treated or infected for 20 hours with *Salmonella*, either left at 37°C (control) or after heat-shock for 1 hour at 46°C. Cells were stained with anti-TIAR antibody (red channel).

The interplay between viruses and RNA granules, specifically PBs and SGs, has been characterized, whereas effects of bacterial infections on these granules have not been studied. To test whether bacterial infection impacts on PBs, HeLa cells – an epithelial cell line widely used for *Salmonella* studies – were infected with GFP-expressing bacteria for twenty hours, and then processed for immunofluorescence using antibodies which recognize the PB marker DDX6 (a.k.a. Rck/p54). Visual inspection of the immunofluorescence images indicated similar PB numbers in mock-treated cells, and in those cells of the infected samples that had not internalized the bacteria; whereas the subpopulation of host cells invaded by *Salmonella* showed reduction in PB number (Figure 1A).

For quantification of PB number per cell in the different cell populations (mock-treated, *Salmonella* negative and *Salmonella* positive), we used automated image acquisition and analysis (see methods and Figure S1) to both distinguish between *Salmonella* positive and negative cells, and count PBs per cell in a systematic and unbiased way, for a significant number of cells (∼1,000 cells per experimental condition). Figure 1B shows that the average number of PBs per cell was reduced by approximately twofold in cells containing *Salmonella*, as compared to mock-treated (p<0.001) or non-invaded cells (p<0.01). Similar results were obtained using anti-GW182 patient autoimmune serum (IC-6 serum), which recognizes the two well characterized PB proteins, GW182 and Ge-1 (Figure S2A; [26]). Thus, successful bacterial infection negatively impacts on the number of PB per cell.

To determine whether infection impairs the formation of new PBs, or induces the disassembly of existing PBs, we used the protein synthesis inhibitor puromycin. Puromycin is well-established to increase both PB size and number, by causing premature polypeptide chain termination and polysome disassembly [27]; this in turn increases the pool of non-translating mRNPs which are essential for PB assembly [3], [28], [29]. HeLa cells were infected for twenty hours, followed by one hour of puromycin treatment. Puromycin invariably stimulated PB formation in all three investigated samples (Figure 1A-B), arguing that infection is inducing PB disassembly rather than inhibiting their formation.

To determine whether *Salmonella* induces PB disassembly in other cell types as well, we infected A431 cells, a human epithelial cell line, and RAW 264.7 cells, a murine cell line with macrophage-like characteristics. Intriguingly, PB dispersion was also observed in A431 cells, while infection had no impact on PB integrity in RAW 264.7 cells (Figure S2B). Overall, these results show that *Salmonella* infection causes PB disassembly, in a cell-type specific manner.

Considering that PBs and SGs share some protein and mRNA components and that both are involved in regulating cytoplasmic degradation and/or storage of mRNAs, we also tested *Salmonella* effects on SG assembly and integrity. In mock-treated cells, the RNA binding protein TIAR is distributed throughout the cell, and it partially redistributes to cytoplasmic SGs upon heat-shock (Figure 1C), as reported previously [30]. However, we did not observe relocalization of the TIAR protein at early or late times post-infection (2, 4 and 20 hours p.i.; Figure 1C and Figure S2C), indicating that *Salmonella* infection does not induce SG formation. In support of this observation, the localization of Ataxin-2 – another well characterized SG marker [31] – did not change either (data not shown). Likewise, *Salmonella* failed to inhibit SG formation induced by heat-shock (Figure 1C). Thus, interference of *Salmonella* with the formation of mRNA granules is specific to PBs.

### RNA synthesis and microRNA activity remain intact upon *Salmonella* infection

**Figure 2:**
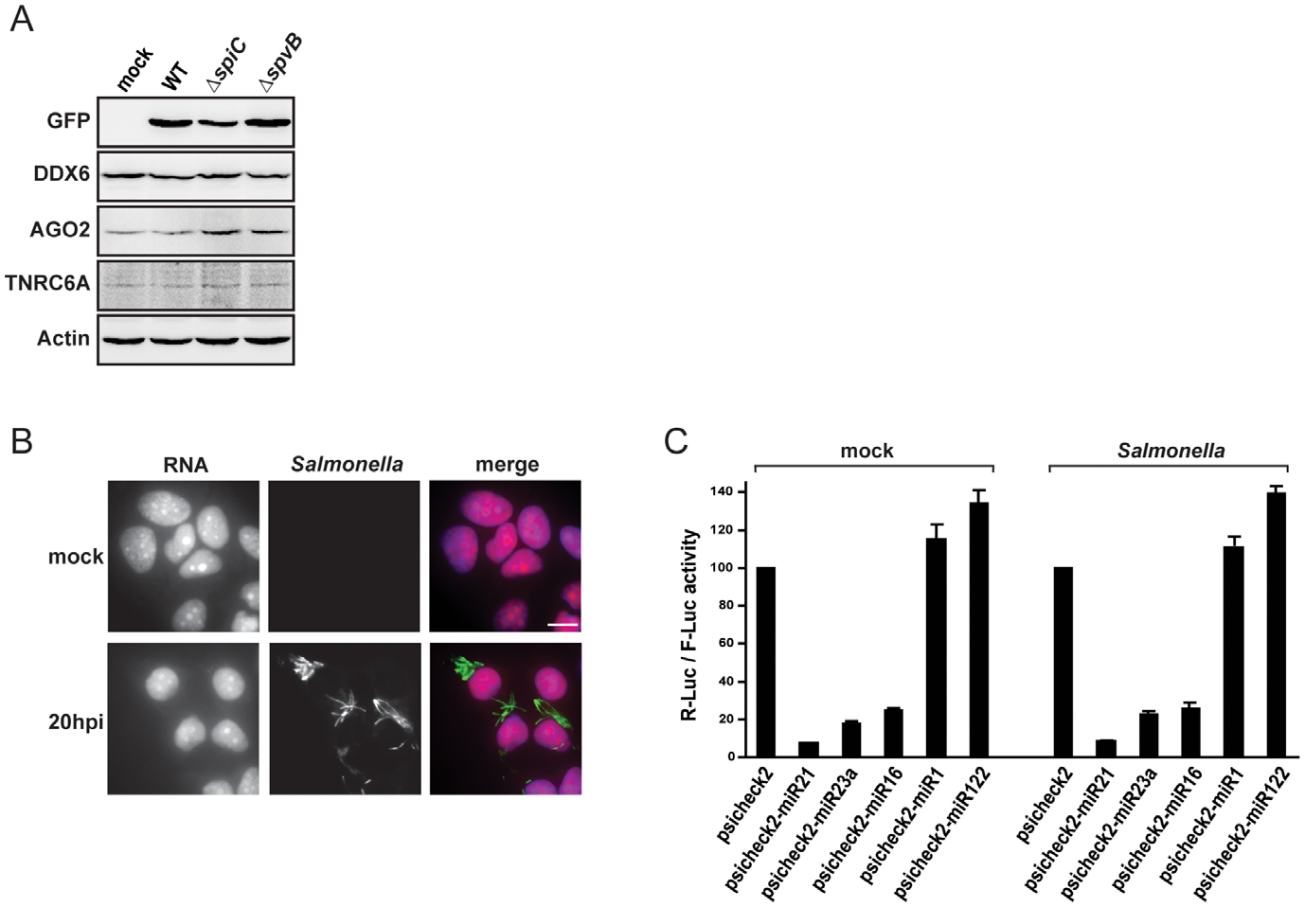
*Salmonella* infection does not interfere with host cell RNA synthesis and microRNA silencing pathways. (A) Western-blot analysis of DDX6, AGO2 and GW182 protein levels in HeLa cells mock-treated or infected for 20 hours with *Salmonella* WT or ΔSpiC and ΔSpvB mutant strains. GFP signal is shown to confirm the presence of intracellular bacteria. (B) Analysis of host cell RNA synthesis in mock-treated and *Salmonella* infected cells (20 hours p.i.), through the incorporation of EU in newly transcribed RNA, followed by the detection using Alexa Fluor 594 azide (red channel), as described in Materials and Methods. *Salmonella* was stained with anti-LPS antibody (green channel). Scale bar, 10 µm. (C) HeLa cells were transfected with the psiCHECK2 empty plasmid or with psiCHECK2 plasmids carrying binding sites for the indicated microRNAs. Twenty-four hours after transfection, cells were mock-treated or infected with *Salmonella*. Luciferase activities were measured 20 hours after infection. Renilla luciferase (R-Luc) activity was normalized to that of the Firefly control (F-Luc) and normalized R-Luc activities of cells transfected with the empty vector were set to 100. Error bars represent standard deviations from three independent experiments.

Given that *Salmonella* causes dramatic gene expression changes in infected host cells, we asked whether PB disassembly might simply result from reduced levels of cellular proteins that are essential for PB integrity. Specifically, we determined protein levels of DDX6, a DExH/D-box RNA helicase with multiple roles in mRNA metabolism, and of AGO2 and TNRC6A, two key proteins of the microRNA pathway. However, Western-blot analysis of extracts of *Salmonella* infected HeLa cells revealed no expression changes of the DDX6, TNRC6A and AGO2 proteins, as compared to mock-treated cells (Figure 2A).

PB maintenance is well-established to depend on the presence of mRNPs that are committed to, or are undergoing, degradation. Therefore, we tested if *Salmonella* infection inhibits RNA synthesis in the host, which then would cause PB disassembly due to a reduction of the pool of non-translating cytoplasmic mRNAs. We assayed incorporation of 5-ethynyluridine (EU) into newly transcribed RNA, which is then detected using fluorescent azides [32]. Both mock and *Salmonella* infected cells (20 hours p.i.) show a comparably strong nuclear and cytoplasmic RNA staining (Figure 2B), arguing that infection does not abrogate host cell RNA synthesis. In contrast, control treatment of cells with actinomycin D, an inhibitor of RNA polymerase, did yield the expected strong decrease of EU staining throughout the cell (data not shown and [32]).

In addition to RNA synthesis, an intact microRNA pathway is important for PB maintenance, and was thus considered as another potential target of infection. To test whether *Salmonella* decreased global microRNA activity, HeLa cells were transfected with reporter vectors containing microRNA binding site downstream of a renilla luciferase ORF, in a vector that also expresses firefly luciferase as internal reference. Three reporter plasmids contained microRNA binding sites for miR-21, miR-23a or miR-16, all of which are highly expressed in HeLa cells yet not regulated by *Salmonella* infection (Schulte et al., in revision). Figure 2C shows that the reporters were strongly down-regulated in HeLa cells (between 4-fold and 13-fold repression), but invariably so in both mock-treated and *Salmonella*-infected cells, arguing that the bacteria do not generally impair microRNA activity. As additional controls, reporters containing binding sites for microRNAs that are not expressed in HeLa cells (miR-1 and miR-122) showed neither basal nor *Salmonella*-specific regulations. Altogether, these results argued against the possibilities that *Salmonella* induces PB disassembly via interference with general functions such as mRNA synthesis or microRNA-mediated gene silencing.

### The SPI-2 T3SS is required for P-body disassembly

**Figure 3:**
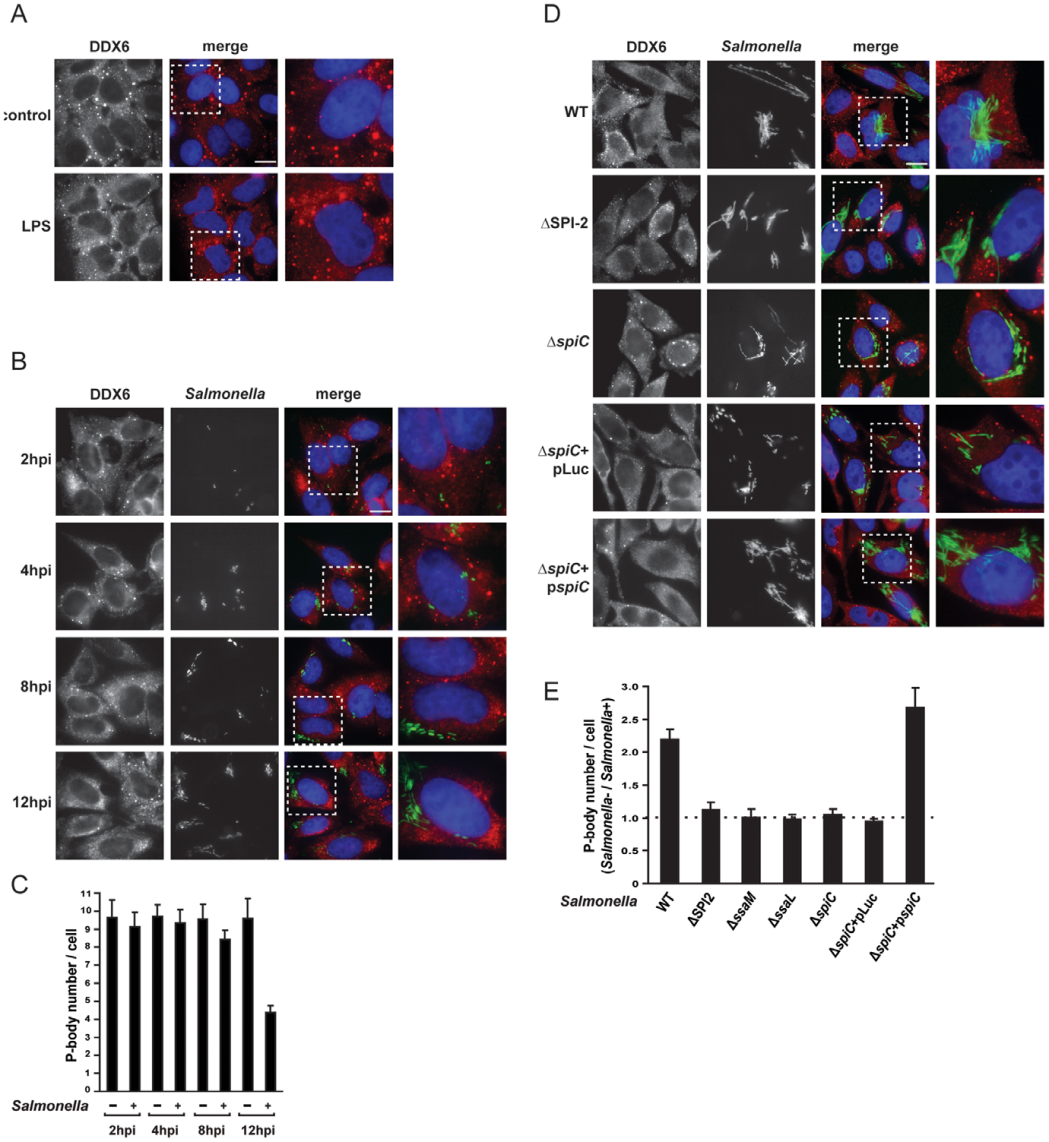
*Salmonella* induced PB disruption is dependent on the activation of the SPI-2 T3SS. (A) HeLa cells were mock-treated or treated with LPS (1 µg/ml) for 20 hours. Cells were stained with anti-DDX6 antibody. Scale bar, 10 µm. The region highlighted by a white square is enlarged on the rightmost panel. (B) HeLa cells were infected with *Salmonella* for 2, 4, 8 and 12 hours as indicated, and then processed for PB detection using anti-DDX6 antibody. (C) Average number of PBs per cell in the population of cells in which *Salmonella* was not internalized (*Salmonella* −) and in cells in which the bacteria were internalized (*Salmonella* +), at the indicated times post-infection. Mean values ± standard deviations from at least three independent experiments are shown. (D) HeLa cells were infected with wild-type *Salmonella* or with the indicated mutant strains and collected at 20 hours p.i. PBs were detected using anti-DDX6 antibody (red channel). (E) Ratio of the average number of PBs per cell between the cell population in which bacteria where not internalized (*Salmonella* −) and the infected cells (*Salmonella* +) for wild-type and mutant *Salmonella* strains. Mean values ± standard deviations from at least three independent experiments are shown.

We considered the possibility that the observed PB disassembly could be associated with sensing of bacterial lipopolysaccharide (LPS) during the infection process. However, this was ruled out by the observation that incubation of HeLa cells with purified *Salmonella* LPS did not affect PB integrity (Figure 3A).

More generally, *Salmonella* infection is a sequential process, initiated by *Salmonella* inducing its own uptake into host cells via the effects of SPI-1 effector proteins, and followed by intracellular manipulation of host cell functions by the SPI-2 T3SS. A detailed analysis of the kinetics of PB disassembly upon *Salmonella* infection revealed that PB integrity is not affected at early and intermediate times of infection (2, 4 or 8 hours p.i.; Figure 3B–C) when the SPI-1 T3SS plays a major role. PB disassembly became apparent only at later times post-infection (12 and 20 hours p.i.; Figure 3B–C), indicating that it might be associated with the later activity of the SPI-2 T3SS which is highly expressed after bacterial enclosure in the SCV. In favour of this hypothesis, infection of HeLa cells with a *Salmonella* mutant deleted for the SPI-2 region (ΔSPI-2) was observed not to induce PB dispersion. Indeed, quantification revealed a ratio of ∼1.0 between the average number of PBs in cells without internalized bacteria (*Salmonella*−) and those successfully infected with the ΔSPI-2 mutant (*Salmonella*+); in other words, the same number of PBs per cell in either cell population (Figure 3E). In contrast, infection with wild-type *Salmonella* resulted in a ratio of 2.2 (Figure 1A, 3E).

The SpiC/SsaM/SsaL complex is a key component of functional SPI-2 T3SS, and regulates the secretion of both effector and translocon proteins [33], [34], the latter of which form a pore in the host membrane vacuole. To confirm the relationship between SPI-2 dependent secretion and PB disassembly, HeLa cells were infected with *Salmonella* Δ*ssaM*, Δ*ssaL* or Δ*spiC* mutants. Similarly to the ΔSPI-2 strain, infection with any of these three mutants failed to impact on PB integrity (Figure 3D and Figure S3). As expected, complementation of the Δ*spiC* mutant with a plasmid containing the wild-type *spiC* allele (Δ*spiC*+p*spiC*) rescued the PB disassembly phenotype (Figure 3D–E). Collectively, this data implicates the activity of the SPI-2 T3SS in PB dispersion.

### *Salmonella* effector protein SpvB is involved in PB disassembly during infection

**Figure 4:**
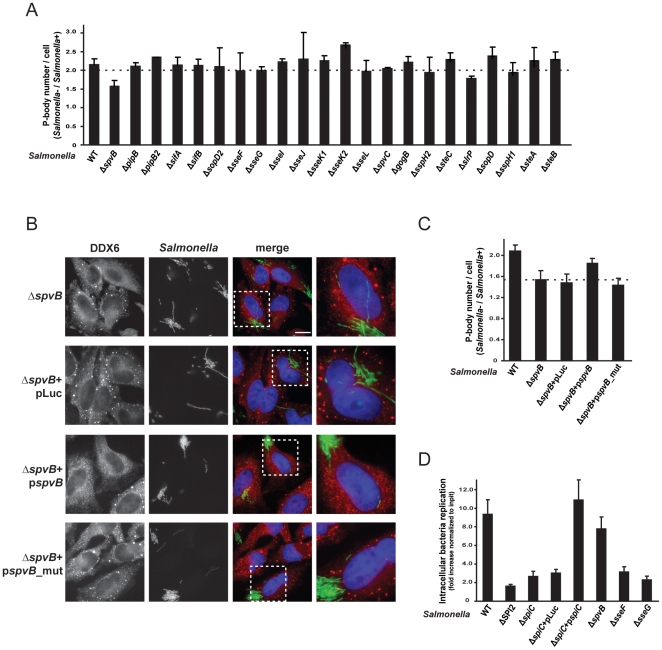
SpvB effector protein is involved in PB disassembly induced by *Salmonella* infection. (A) HeLa cells were infected with wild-type or single mutants of all *Salmonella* effector proteins. The ratio of the average number of PBs per cell, between the cell population in which bacteria where not internalized (*Salmonella* −) and the infected cells (*Salmonella* +), was calculated. Mean values ± standard deviations from at least three independent experiments are shown. Representative images of cells infected with the different mutants are shown in Figure S4. (B) HeLa cells were infected with wild-type *Salmonella* or with the indicated mutant strains. Twenty-hours post-infection cells were processed for immunofluorescence and PBs were detected using anti-DDX6 antibody (red channel). Scale bar, 10 µm. The region highlighted by a white square is enlarged on the rightmost panel. (C) Ratio of the average number of PBs per cell between the cell population in which bacteria where not internalized (*Salmonella* −) and the infected cells (*Salmonella* +) for wild-type and mutant *Salmonella* strains. (D) Intracellular replication assays were performed in HeLa cells with the indicated strains. Values indicate the fold increase, calculated as the ratio between the intracellular bacteria at 20 hours p.i. and the input bacteria used for infection. Error bars represent standard deviations from three independent experiments.

There are over twenty *Salmonella* proteins that are translocated by the SPI-2 T3SS into the host cell cytoplasm, and many of them act in concert to subvert the host cell cytoskeleton, signal transduction pathways, membrane trafficking and pro-inflammatory responses [21], [22], [23]. To determine which of these proteins might promote PB dispersion, we generated a collection of effector gene mutants and screened for their effect on PB integrity. Most of the tested mutants induced PB disassembly to the same extent as did wild-type *Salmonella* (Figure 4A and Figure S4). However, a Δ*spvB* mutant showed significantly lower PB disruption (Figure 4A–C), resulting in a *Sa* lmonel *la*−/*Salmonella*+ PB ratio of approximately 1.5 (p?0.001). Importantly, the Δ*spvB* strain replicates almost as efficiently as the wild-type strain (Figure 4D), arguing against the possibility that a lower replication rate impairs its ability to cause PB disruption. Along the same line, we observed *Salmonella* mutants which infect HeLa cells with efficiency comparable to that of the Δ*spiC* mutant but still induce PB disassembly (e.g. Δ*sseG* and *sseF*; Figure 4D), reinforcing the notion that PB dispersion is not generally coupled to bacterial replication or load. Complementation of the Δ*spvB* mutant with a plasmid carrying the *spvB* gene (Δ*spvB*+p*spvB*) restored PB disassembly, whereas the parental cloning vector (Δ*spvB*+pLuc) did not (Figure 4B–C).

The only previously known function of the *Salmonella* SpvB protein is an ADP-ribosyl transferase activity on monomeric actin as the primary substrate, blocking actin polymerization to F-actin filaments during infection [35], [36], [37], [38], [39]. This catalytic activity is abrogated by mutation of two conserved glutamate residues to aspartates (E538D and E540D) of SpvB [35]. Similarly, we observed that mutation of these residues in our *spvB* complementation plasmid rendered it unable to complement the Δ*spvB* strain for PB disassembly (Figure 4B and C), indicating that *Salmonella* infection might interfere with PB integrity by perturbing the actin cytoskeleton. However, treatment of HeLa cells with cytochalasin D, a potent inhibitor of actin polymerization, had no effect on PB integrity (data not shown), arguing that SpvB targets PB integrity in a process unrelated to its actin depolymerizing activity.

### PB disassembly induced by bacterial infection is not restricted to *Salmonella*

**Figure 5:**
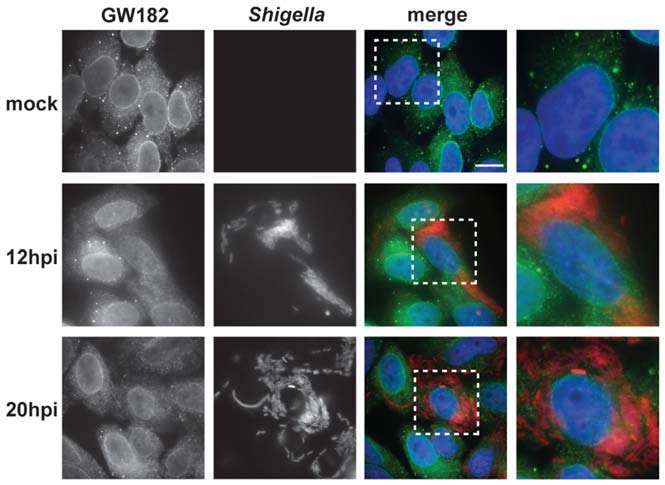
*Shigella* infection also induces PB disassembly. HeLa cells were mock-treated or infected with *Shigella* for 12 and 20 hours. PBs and *Shigella* were detected using anti-GW182 (green channel) and anti-*Shigella* (red channel) antibodies, respectively. Scale bar, 10 µm. The region highlighted by a white square is enlarged on the rightmost panel.

To establish whether PB disassembly could be a more general characteristic of bacterial pathogens, we analyzed the integrity of PBs in cells upon invasion by *Shigella flexneri*, an enteroinvasive pathogen that like *Salmonella* has epithelial cells as primary target [40]. Similar to *Salmonella*, *Shigella* also dispersed PBs after internalization (Figure 5). This was most evident at intermediate and late times post-infection (6, 12 and 20 hours p.i.; Figure 5 and data not shown). Thus, PB disassembly induced by bacterial infection is not restricted to *Salmonella*, indicating that this phenomenon likely has biological significance to the life cycle of different bacteria.

## Discussion

Bacterial pathogens are endowed with extraordinarily abilities to subvert host cell functions as they initiate and establish an infection. Extensive work on the model pathogen *Salmonella* has revealed a plethora of protein-based signaling cascades and structural functions as targets of T3SS-secreted virulence factors, yet whether and how bacteria interfere with RNA-related processes in their hosts has only begun to be addressed [41]. The present study is the first to address the effect of bacterial infection on P-body integrity. Our results show that *Salmonella* infection induces PB disassembly in epithelial cells, and implicates the SPI-2 encoded T3SS and the associated SpvB effector as one factor in this process. This effect is not restricted to a single bacterial pathogen, since *Shigella* infection also induces PB disruption.

Previous reports have demonstrated that although PBs are not required for silencing, PB formation can be prevented by blocking the microRNA pathway, either at the level of microRNA biogenesis (e.g., by depleting Dicer or Drosha) or at the silencing step (e.g., by AGO or GW182 knockdown; [3], [4], [42]). Using deep sequencing of the small RNA population of cells infected with *Salmonella*, we have shown that bacterial infection induces the regulation of a limited number of microRNAs (Schulte et al., in revision). Specifically, we found that most microRNAs of HeLa cells were unaffected by *Salmonella*, with only the let-7 family of microRNAs being twofold downregulated. Consistent with a limited effect on microRNA pathways, reporter vectors containing microRNA binding sites were here observed to be invariably regulated during *Salmonella* infection. A recent study in human macrophage-like THP1 cells revealed that LPS stimulation increases of the number of PBs per cell, and attributed this effect to increased miR-146 expression [43]. However, stimulation of HeLa cells with LPS does not affect PB number or miR-146 levels (Schulte et al., in revision), likely due to the fact that the LPS sensing components TLR2 and the TLR4 co-factor MD-2 are not expressed in HeLa cells [44], [45]. Collectively, these results argue that the *Salmonella*-induced PB disruption is not a simple consequence of impaired microRNA biogenesis and activity.

Interference with key components of PBs, either proteins or RNA, can also result in PB dispersion. We provide evidence that the PB disassembly upon bacterial infection is unrelated with an effect on the general mRNA synthesis. Additionally, we show that the levels of crucial PB proteins (DDX6, AGO2 and TNRC6A) remain unchanged during infection. Likewise, PB formation remains intact in infected cells treated with the protein synthesis inhibitor puromycin, arguing that *Salmonella* infection does not block PB formation, but rather disassembles existing PBs. This again supports the notion that essential PB biogenesis components are not directly targeted by *Salmonella* infection. Intriguingly, however, we observe that the relative increase in PB formation after puromycin treatment is strongest in *Salmonella*-containing cells, as if the infection limits the pool of translationally repressed cytoplasmic mRNAs required for PB formation.

Analysis of PB integrity over the course of infection suggests that PB disruption occurs late, and argues that bacterial internalization, per se, is not sufficient for the observed phenomenon. Moreover, our results demonstrate that the *Salmonella* SPI-2 T3SS is essential for PB dispersion. That is, there was no PB disassembly in cells infected with *Salmonella* mutants deprived of either the complete SPI-2 island or individual components of the SpiC/SsaM/SsaL complex required for SPI-2 dependent translocon assembly and effector secretion [33], [34]. Although a matter of controversy, the SpiC protein was also reported as being translocated by the SPI-2 T3SS, and to interfere with endosomal trafficking [46]. However, given that overexpression of SpiC in HeLa cells had no effect on PB integrity (data not shown), SpiC function as a secreted effector protein is unlikely involved in PB dispersion.

Our candidate approach has identified the SPI-2 effector SpvB as a potential mediator of PB disruption; of all mutants tested, the *Salmonella* Δ*spvB* strain was consistently impaired in PB disassembly, relative to the wild-type strain. The plasmid-encoded SpvB protein has been known to induce depolymerization of the actin cytoskeleton at late stages of infection via its ADP-ribosyl-transferase activity [35], [36], [37], [38], [39]. At first glance, our result showing that a catalytically inactive ADP-ribosyl-transferase mutant of SpvB is also unable to disperse PBs lends itself for the straight-forward conclusion that SpvB's actin-depolymerizing activity critically contributes to PB dispersion by *Salmonella*. However, while previous studies showed that microtubule disruption increases in the average number of PB per cell, interference with the actin cytoskeleton has little effects on PB assembly [47], [48]. Moreover, cytochalasin D – an inhibitor of actin polymerization – was here found to have no impact on PB disruption. In other words, although the same region of SpvB is critical for both processes, PB disruption cannot be a simple consequence of SpvB-mediated actin depolymerization. Interestingly, the N-terminus of SpvB has homology with the TcaC protein of *Photorhabdus luminescens*, a secreted insecticidal toxin that acts through an unknown mechanism and causes cytopathology [49]. Thus, is the observed PB disruption a consequence of a more general cytotoxic effect? Rather not: when we overexpressed SpvB alone in HeLa cells from a strong eukaryotic promoter, transfected cells did show considerable morphological changes and reduction of F-actin staining yet PB numbers remained unaffected (data not shown).

Whilst the precise mechanism of SpvB action is yet to be unraveled, its impact on PBs is likely part of a multi-factorial process. First, although the Δ*spvB* strain was consistently impaired in PB disruption, the lack of SpvB alone does not match the stronger impairment of strains deficient in SPI-2 activity, suggesting redundant function of another factor(s) in the same pathway. Second, expression of SpvB in HeLa cells does result in actin depolymerization yet is not sufficient to disperse PB, suggesting that SpvB either needs the activity of other bacterial or cellular proteins either translocated by *Salmonella* or induced in the host by the infection, respectively. Third, the *spvB* gene is induced rather early during infection [50], [51], whereas PB disruption is a late process. Moreover, although *Shigella* also induces PB dispersion, none of the known secreted *Shigella* effectors shows obvious homology with SpvB, and none of the characterized effectors in Shigella has been shown to have ADP-ribosyl-transferase activity. Thus, future work to shed light on the mechanisms of PB dispersion will require a comprehensive analysis of protein abundance changes and putative SpvB interaction partners during *Salmonella* infection.

Recent work in viral infection systems, specifically infection with Dengue and West Nile viruses, has shown that the PB number per cell decreases at late times post-infection, but no insights regarding the proteins, pathways or relevance of this phenotype were obtained [17]. Interestingly, in the case of SGs and poliovirus infection, which early on induces the formation of these granules, but later loses this ability, a viral protease was shown to cleave G3BP, a critical SG component, and by this mechanism block SG formation and increase viral replication [52]. Undoubtedly more research is necessary to determine what is the mechanism of PB disassembly induced by bacterial and viral infection, and if this phenotype is a requirement and/or a consequence of infection. Nevertheless, our study identifying PB integrity as a novel target of bacterial infection, following similar observations for some viruses, highlights the physiological importance of PB disruption by pathogens.

## Materials and Methods

### Bacterial strains

*Salmonella* enterica serovar Typhimurium strain SL1344 constitutively expressing GFP from a chromosomal locus (strain JVS-3858) is referred to as wild-type (WT) throughout this work. The mutant strains were constructed from a non-tagged strain (JVS1574) using the lambda red recombinase method [53]. A complete list of the primers used to construct and verify the mutant strains is provided in Table S1. The collection of mutant strains was tagged with GFP by phage P22 transduction of the respective strain with a lysate of strain JVS-3858. In the complemented strains, the proteins SpiC and SpvB are expressed from the pXG0 low copy plasmid, under the control of the respective promoter. The primers used for cloning are indicated in Table S2. *Shigella flexneri* serotype 5 strain M90T was kindly provided by Anna Zumsteg (Max Planck Institute for Infection Biology, Berlin Germany).

### Cell culture, heat-shock, LPS and puromycin treatment

HeLa (ATCC, CCL-2), RAW 264.7 (ATCC, TIB-71) and A431 (ATCC, CRL-1555) cells were grown in RPMI 1640 (GIBCO) supplemented with 10% fetal calf serum (Biochrom), 2 mM L-glutamine (GIBCO), 1 mM sodium-pyrovate (GIBCO) and 0.5% β-mercapto-ethanol (GIBCO) in a 5% CO_2_, humidified atmosphere, at 37°C.

SG formation was induced by heat-shock, by incubating the cells 1 hour at 46°C in a 5% CO_2_, humidified atmosphere. For LPS treatment, cells were incubated with 1 µg/ml purified LPS from *Salmonella* enteric serovar Typhimurium (Sigma) for 20 hours. Puromycin treatment was performed by incubating the cells for 1 hour with 100 µg/ml puromycin (Calbiochem).

### Bacterial infection and quantification of *Salmonella* intracellular replication

Twenty-four hours prior to infection, 2.0×10^5^ of cells were seeded into Lab-Tek II chamber slides (Nalge Nunc; for immunofluorescence) or 12-well plates (for protein samples and intracellular replication assays).

Overnight cultures of *Salmonella* or *Shigella* were diluted 1∶100 in fresh L-broth medium and grown aerobically until OD 2. Bacteria were harvested by centrifugation and resuspended in complete RPMI medium. For immunofluorescence and intracellular replication assays cells were infected at MOI of 5, and for preparation of protein extracts at an MOI of 50. After addition of bacteria, cells were centrifuged at 250×g for 10 min at room temperature followed by 20 min incubation in 5% CO_2_, humidified atmosphere, at 37°C. Medium was then replaced with complete RPMI containing gentamicin (50 µg/ml) to kill extracellular bacteria. Following 30 min incubation, the medium was replaced with new complete RPMI containing 10 µg/ml gentamicin for the rest of the experiment. Unless otherwise indicated, cells were collected 20 hours after infection.

To quantify *Salmonella* intracellular replication, infected HeLa cells were thoroughly washed with phosphate-buffered saline (PBS) and lysed with PBS containing 0.1% Triton-X-100. Samples were then serially diluted in PBS and plated on LB plates. The number of colonies formed from the recovered bacteria was compared to that obtained from the input bacteria used for infection.

### Immunofluorescence and total RNA staining

Cells were fixed with 4% paraformaldehyde for 15 min, permeabilized with 0.5% Triton X-100 in PBS during 5 min, followed by 30 min blocking in 1% bovine serum albumin (BSA). Cells were then stained for 1 hour at room temperature with the following primary antibodies diluted in blocking solution: rabbit anti-DDX6 (Bethyl; 1∶500), human anti-GW182 (IC-6 serum, kindly provided by Marvin J. Fritzler, University of Calgary, Calgary, Canada; 1∶5000), mouse anti-TIAR (BD Transduction Laboratories; 1∶200), or rabbit anti-*Shigella* flexneri (Axell; 1∶200). Cells were washed with PBS and incubated for 1 hour with the respective secondary antibodies conjugated to Alexa Fluor 488 or 594 (Invitrogen; 1∶500).

For staining of total RNA the Click-iT RNA imaging kit (Invitrogen) was used according to the manufacturer's instructions. Briefly, mock-treated or *Salmonella* infected cells were labelled with 0.5 mM 5-ethynyl uridine (EU) for the 20 hours of infection, cells were then fixed, permeabilized as described above, and the Click-iT reaction cocktail containing the Alexa Fluor 594 azide was added for 30 min. Cells were then washed and processed for immunofluorescence using mouse anti-*Salmonella* LPS (Abcam: 1∶1000) and Alexa 488 labelled goat anti-mouse (Invitrogen; 1∶500), as described above.

In all cases, cells were stained with Hoechst 33342 (Invitrogen; 1∶5000) diluted in PBS and mounted in Vectashield mounting medium (Vector Laboratories). Images were acquired using a Leica DMR microscope equipped with a Nikon Dxm1200F digital camera.

### P-body quantification

PB quantification was performed at the ICGEB High-Throughput Screening Facility (http://www.icgeb.org/high-throughput-screening.html). Image acquisition was performed using an ImageXpress Micro automated high-content screening microscope (Molecular Devices) equipped with a 40x objective; a total of 48 fields were acquired from each coverslip, which corresponds to an average of ca. 1000 cells imaged and analysed per experimental condition and replicate. Automated image analysis was then performed in two sequential steps using the MetaXpress software (Molecular Devices). Firstly, nuclei were segmented and cells were classified as positive or negative for *Salmonella*, depending on the total area of *Salmonella* staining (GFP, green channel) above the background, and subsequently the number of PB was quantified for each cell (red channel); analysis were performed using the “Multi Wavelength Cell Scoring” and “Transfluor” application modules built in MetaXpress. *Salmonella* and PBs are assigned to a specific cell based on their proximity to neighboring nuclei. Results were then combined, on a cell by cell basis, using AcuityXpress software (Molecular Devices) and the average number of PB per cell was calculated for the population of cells positive or negative for intracellular *Salmonella*.

### Whole-cell protein extracts and Western-blot

Cell pellets were resuspended in 1x sample loading buffer (Fermentas), and protein samples were separated in a 10% SDS-PAGE, followed by Western-blotting. The following antibodies were used: mouse anti-GFP (Roche; 1∶1000), rabbit anti-DDX6 (Bethyl; 1∶1000), rat anti-AGO2 (kindly provided by Gunter Meister, Max Planck Institute of Biochemistry, Martinsried, Germany; 1∶2000), mouse anti-GW182 (Santa-Cruz; 1∶50) and mouse anti-β-actin (Applied Biosystems; 1∶2000). Bound primary antibodies were detected with horseradish peroxidase-coupled secondary antibodies (Amersham; 1∶5000) followed by detection using Western Lightning reagent (PerkinElmer). Signals were detected with a Fuji LAS-3000 CCD camera.

### Transfection and Luciferase assays

microRNA reporter plasmids containing binding sites for miR-21, miR-23a, miR-16, miR-1 or miR-122 downstream of the renilla luciferase coding sequence of the psiCHECK2 luciferase reporter vector (Promega) were kindly designed, cloned and provided as a gift for research purposes by Thermo Fisher Scientific Research and Development scientists.

For the transfection experiments 5.0×10^4^ HeLa cells were seeded onto 12-well plates 24 hours prior to transfection. Reporter vectors (1 µg) were transfected using Lipofectamine 2000 (Invitrogen), according to the manufacturer's instructions. Twenty-four hours after transfection cells were mock-treated or infected with *Salmonella* as described previously, and cells were collected 20 hours later. Firefly and renilla luciferase activities were measured on a Wallac 1420 Victor3 multilabel counter (PerkinElmer) using beetle-Juice and renilla-juice (PJK GmbH).

### Statistics

The mean ± standard deviations for at least three independent experiments is shown in figures, and p values were calculated using One-way ANOVA and Tukey's Multiple Comparison Test (Graph Pad, Prism Software). A p value of less than 0.05 was considered to be statistically significant.

## Supporting Information

Figure S1 Overview of the procedure to quantify the number of PBs in *Salmonella* infected cells. (A) Low-resolution montage showing 48 image fields acquired at a 40x magnification. (B) High-resolution image of *Salmonella* infected cells. Cell nucleus was stained with Hoechst 33342 (blue), *Salmonella* was detected in the green channel and PBs were detected using anti-DDX6 antibody (red channel). A total of 48 fields were imaged per coverslip, which corresponds to approx. 1000 cells per experimental condition. (C) Original images and results from the image segmentation showing *Salmonella* positive cells (green cells, bottom *Salmonella* pannel) and PBs (rightmost bottom panels).
(TIF)

Figure S2 PB disruption induced by *Salmonella* infection is cell-type dependent. (A) HeLa cells were mock-treated or infected with *Salmonella* for 20 hours. PBs were detected using anti-GW182 (red channel). Scale bar, 10 µm. The region highlighted by a white square is enlarged on the right side of the panel. (B) RAW264.7 and A431 cells were mock-treated or infected with *Salmonella* for 20 hours. PBs were stained with anti-DDX6 antibody (red channel). (C) SG formation was tested in HeLa cells infected for 2 and 4 hours with *Salmonella*. Cells were stained with anti-TIAR antibody (red channel).
(TIF)

Figure S3 SpiC/SsaM/SsaL complex is essential for *Salmonella* interference with PB integrity. HeLa cells were infected with ΔSsaM and ΔSsaL *Salmonella* strains for 20 hours. PBs were stained with anti-DDX6 antibody (red channel). Scale bar, 10 µm. The region indicated by a white square is enlarged on the rightmost panel.
(TIF)

Figure S4 PB integrity is affected by infection with *Salmonella* mutant strains of the tested SPI-2 T3SS dependent effector proteins. HeLa cells were infected with the indicated *Salmonella* mutant strains for 20 hours. PBs were stained with anti-DDX6 antibody (red channel). Scale bar, 10 µm. The region indicated by a white square is enlarged in the panel below.
(TIF)

Table S1 List of the primers used to construct and verify the *Salmonella* mutant strains.
(PDF)

Table S2 List of the primers used for cloning in pXG0-amp.
(PDF)
