# Supplementary figures and images for: A Candidate Approach Implicates the Secreted Salmonella Effector Protein SpvB in P-Body Disassembly
Source: PLoS One. 2011 Mar 1;6(3):e17296. doi: 10.1371/journal.pone.0017296 (PMC3046968; doi:10.1371/journal.pone.0017296)

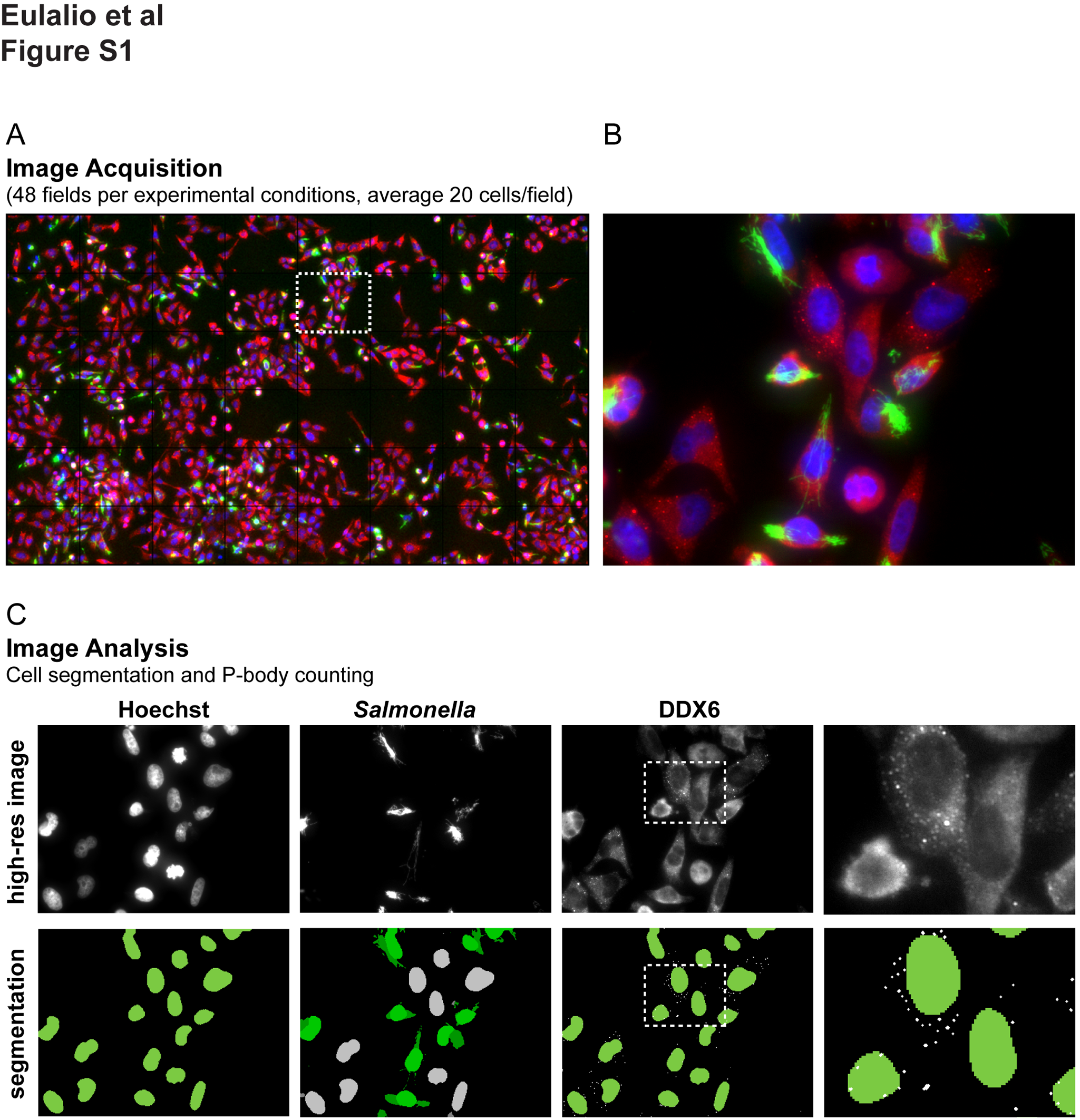

Supplement: Figure S1 — Overview of the procedure to quantify the number of PBs in Salmonella infected cells. (A) Low-resolution montage showing 48 image fields acquired at a 40x magnification. (B) High-resolution image of Salmonella infected cells. Cell nucleus was stained with Hoechst 33342 (blue), Salmonella was detected in the green channel and PBs were detected using anti-DDX6 antibody (red channel). A total of 48 fields were imaged per coverslip, which corresponds to approx. 1000 cells per experimental condition. (C) Original images and results from the image segmentation showing Salmonella positive cells (green cells, bottom Salmonella pannel) and PBs (rightmost bottom panels). (TIF) [file pone.0017296.s001.tif]

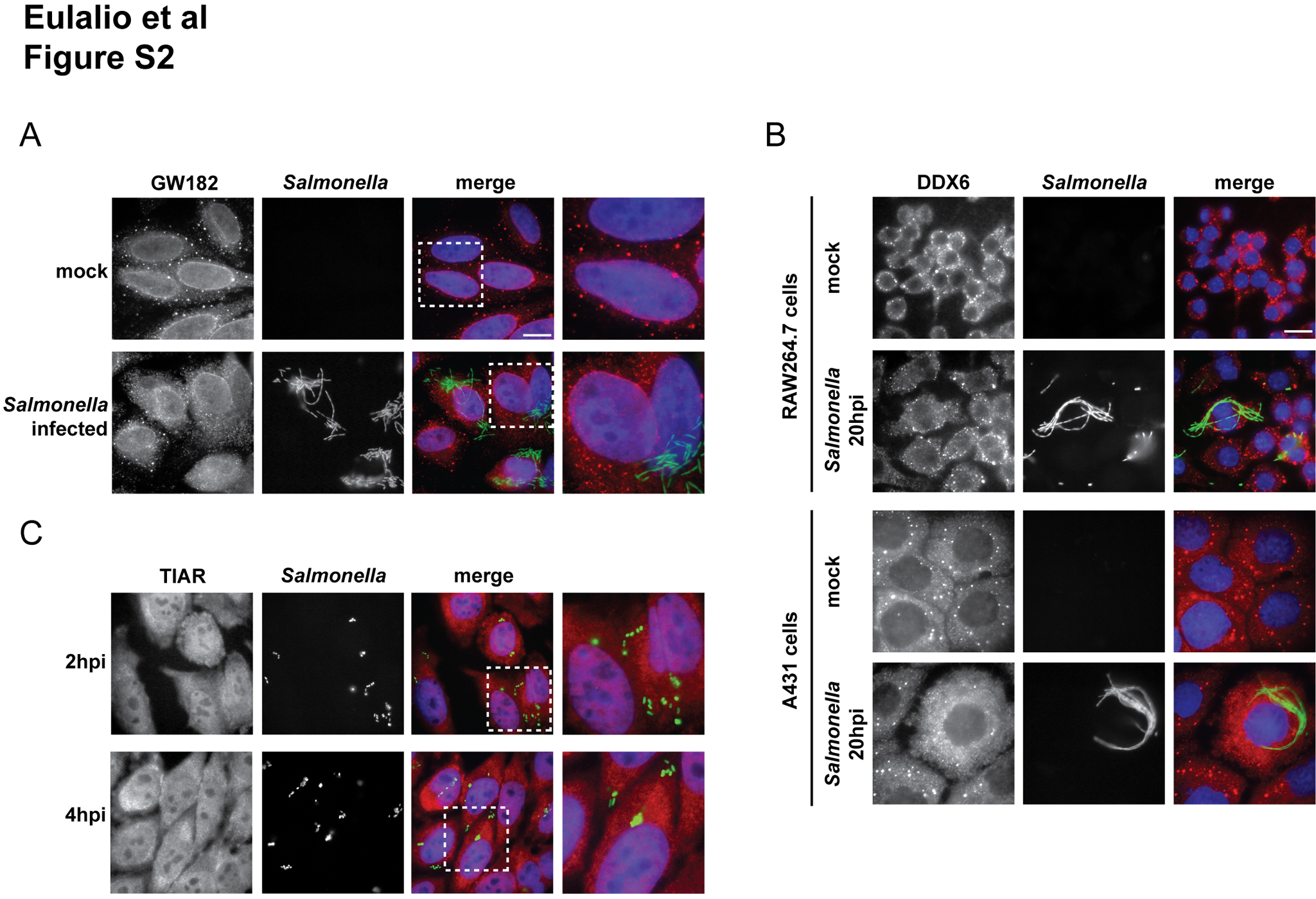

Supplement: Figure S2 — PB disruption induced by Salmonella infection is cell-type dependent. (A) HeLa cells were mock-treated or infected with Salmonella for 20 hours. PBs were detected using anti-GW182 (red channel). Scale bar, 10 µm. The region highlighted by a white square is enlarged on the right side of the panel. (B) RAW264.7 and A431 cells were mock-treated or infected with Salmonella for 20 hours. PBs were stained with anti-DDX6 antibody (red channel). (C) SG formation was tested in HeLa cells infected for 2 and 4 hours with Salmonella. Cells were stained with anti-TIAR antibody (red channel). (TIF) [file pone.0017296.s002.tif]

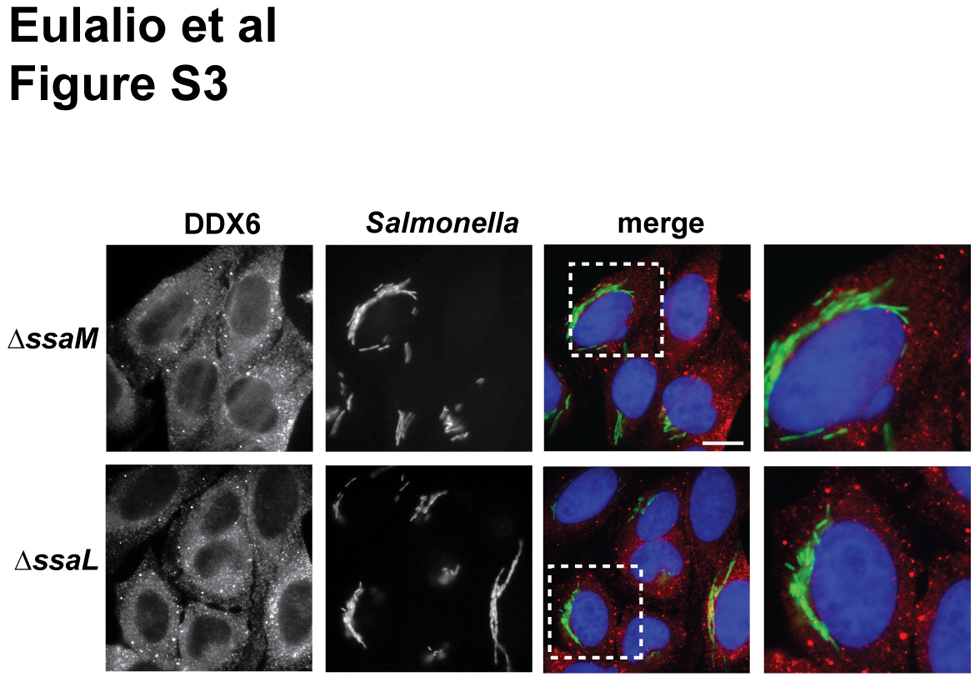

Supplement: Figure S3 — SpiC/SsaM/SsaL complex is essential for Salmonella interference with PB integrity. HeLa cells were infected with ΔSsaM and ΔSsaL Salmonella strains for 20 hours. PBs were stained with anti-DDX6 antibody (red channel). Scale bar, 10 µm. The region indicated by a white square is enlarged on the rightmost panel. (TIF) [file pone.0017296.s003.tif]

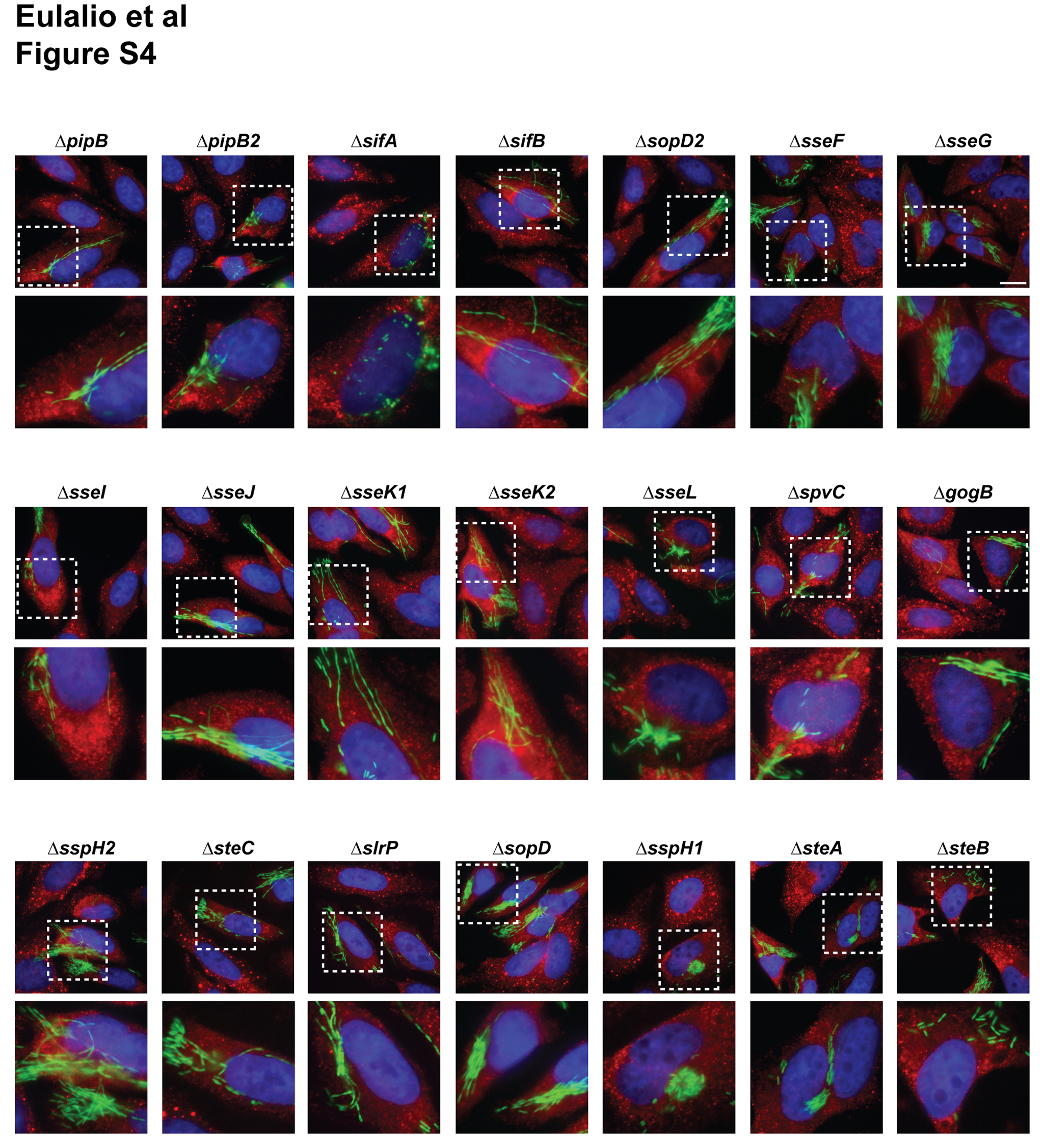

Supplement: Figure S4 — PB integrity is affected by infection with Salmonella mutant strains of the tested SPI-2 T3SS dependent effector proteins. HeLa cells were infected with the indicated Salmonella mutant strains for 20 hours. PBs were stained with anti-DDX6 antibody (red channel). Scale bar, 10 µm. The region indicated by a white square is enlarged in the panel below. (TIF) [file pone.0017296.s004.tif]
