# Supplementary material for: A Candidate Approach Implicates the Secreted Salmonella Effector Protein SpvB in P-Body Disassembly
Source: PLoS One. 2011 Mar 1;6(3):e17296. doi: 10.1371/journal.pone.0017296 (PMC3046968; doi:10.1371/journal.pone.0017296)
Supplement: Table S1 — List of the primers used to construct and verify the Salmonella mutant strains. (PDF) [file pone.0017296.s005.pdf]

Table S1. List of the primers used to construct and verify the *Salmonella* mutant strains.

mutant strain	Strain reference	GFP-tagged strain reference	Oligos for mutant strains	Oligos for verification
ΔsopD	JVS-2890	JVS-8706	JVO-2463- TATCGTCACCACAAAGGATTACCAACCATAAATGTGCTGTGTGTAGGCTGGAGCTGCTTC<br>JVO-2464- TCCGGCAGGCAGCCGGATTTTAAATTGGTTATATTACTGAGGTCCATATGAATATCCTCCTTAG	JVO-1919- CCAGTAACCCACAGCC<br>JVO-1920- TGCATGAAGGGTAATTGTC
ΔpipB	JVS-3453	JVS-8693	JVO-3048- ATGTGACTTGACTCACACCTATAAGGAGTCGGCTCACTTCGTGTAGGCTGGAGCTGCTTC<br>JVO-3049- GTAACGCTATGATTCAGACAAAGCCAACAAGTAATACGCTGGTCCATATGAATATCCTCCTTAG	JVO-3100- TGTCAGATGCCAGACACC<br>JVO-3101- ATCTGTAGGTGACCGGAAG
ΔgogB	JVS-3455	JVS-8689	JVO-3050- ACGATCGGTATTAGGCTAGGTTCTAAATCTTGCCTGAATGGTGTAGGCTGGAGCTGCTTC<br>JVO-3051- ACTCCAATAGGGCTGCTCTATATATAAATATATTAATTGCGGTCCATATGAATATCCTCCTTAG	JVO-3096- CAGTCAGTCCTGACTAAACAGC<br>JVO-3097- CCTTGCCAAACAGATATGTAG
ΔpipB2	JVS-3469	JVS-8694	JVO-3046- ATGGAGCGTTCACTCGATAGTCTGGCTGGTATGGCTAAGTGTAGGCTGGAGCTGCTTC<br>JVO-3047- CTCTCAGCTACTATTCAGTAGCAGATTGTTATTCTTACATGGTCCATATGAATATCCTCCTTAG	JVO-3098- AGCAACGCGTCGTGATAC<br>JVO-3099- ACAGGCACTTATACATCCAGG
ΔsifA	JVS-3471	JVS-8690	JVO-3136- TCCTTACCAACTCCCCAAGGAATACGAAAGAAGCATGGTGGTGTAGGCTGGAGCTGCTTC<br>JVO-3137- TCCGATCCGGTCATATGCGGCATTATTGTGCCTGGCAAGGGTCCATATGAATATCCTCCTTAG	JVO-3138- ACGCTATCACAGACAGTAATGC<br>JVO-3139- AGCCAGGAAGAAGCAAGC
ΔslrP	JVS-3473	JVS-8695	JVO-3054- ATCCTTATCTGTTACTTTAGGTTACGTTCAGATCAGGTAGGTGTAGGCTGGAGCTGCTTC<br>JVO-3055- AGTAGTACGCTCTGCGTCAGAGTCGTCTGCTATTCTTAGGTCCATATGAATATCCTCCTTAG	JVO-3104- AGCGTGTCATGGCAACAG<br>JVO-3105- TCATGAGCGAGCTCACCTC
ΔsifB	JVS-3582	JVS-8696	JVO-3170- AATCACTTGTGGTCTACATTATGCCAATTACTATCGGGAGGTGTAGGCTGGAGCTGCTTC<br>JVO-3171- TCCATACTATTTATGGTGTGATCAACTCTGGTGATGAGCCGGTCCATATGAATATCCTCCTTAG	JVO-3172- ATGTTGACGGTTCAGGAC<br>JVO-3173- AACGAGCCAATTCGTTC
ΔspvB	JVS-3621	JVS-8707	JVO-3187- TCGTCAGACGGCCAGTTTCAGGAGATAGTGTATGTTGATACGTGTAGGCTGGAGCTGCTTC<br>JVO-3188- AGCTGGCTTGCAAAGTCTTGGGATCCACAATTTACAATGCGGTCCATATGAATATCCTCCTTAG	JVO-3189- TAGAGCAGACGCTGTAAGC<br>JVO-3190- TGTGTCAGCAGTTGCATC
ΔspvC	JVS-3622	JVS-8708	JVO-3191- AAACATCCCTCCTTTGAATATTGTAGCTGCTTATGATGGGGTGTAGGCTGGAGCTGCTTC<br>JVO-3192- TTACTGTTCCGTTGCTCCCCAAACCCATACTTACTCTGTCGGTCCATATGAATATCCTCCTTAG	JVO-3193- ATCCATATCGCAAAGGAG<br>JVO-3194- ATGAGCGTTATTCCTTTACG
ΔsseF	JVS-3623	JVS-8699	JVO-3195- AGTCGATGGTAATAGTCCTCCTTCCGATATACAAGCGAAGGTGTAGGCTGGAGCTGCTTC<br>JVO-3196- AGGCAATAATCCAGTACCGCACCTATCGCCGTCAGGCTAACGGTCCATATGAATATCCTCCTTAG	JVO-3197- TCATGGAGTGAGATTCGC<br>JVO-3198- ATCGGCATGAAGTTCATC
ΔsseG	JVS-3624	JVS-8700	JVO-3199- ATTGCCACATCCGGAAACCAATATGGAGAGTGGTAGAATAGTGTAGGCTGGAGCTGCTTC<br>JVO-3200- ATTGCCACATCCGGAAACCAATATGGAGAGTGGTAGAATAGTGTAGGCTGGAGCTGCTTC	JVO-3201- TGGACAGTTCTGATCATACATC<br>JVO-3202- TGTTCTGGCGTTACCTGAG
ΔsseI	JVS-3626	JVS-8701	JVO-3203- TATGCCCTTTCATATTGGAAGCGGATGTCTTCCCGCCATCGTGTAGGCTGGAGCTGCTTC<br>JVO-3204- ATCGCCGGATTGACAGGGTTCTGACAGACGTCCTCCACGGGGTCCATATGAATATCCTCCTTAG	JVO-3205- AGGAAGAGATGATGTCTGTCG<br>JVO-3206- TCTGTCATCTGTGATAGTGTCC
ΔsseK1	JVS-3627	JVS-8703	JVO-3178- TTATGATCCCACCATTAAATAGATATGTTCCCGCGCTTTCGTGTAGGCTGGAGCTGCTTC<br>JVO-3179- TTCAATAGCATGATTATTGCCATTTCCGCTACTGCACATGGGTCCATATGAATATCCTCCTTAG	JVO-3180- TAGCTGACAGCGATTGCAAC<br>JVO-3181- TGCCGTATATCTCCGTTCTG

Δ*sseL*	JVS-3628	JVS-8705	JVO-3211- ACAGCTTATATACAGAAGAGGTGAGCGATGAGGCGCTTACGTGTAGGCTGGAGCTGCTTC<br>JVO-3212- ACCACAGCCGTTGGGTACATTGTTCTGTAAATTCATTTCGGTCCATATGAATATCCTCCTTAG	JVO-3213- TTCGCTACTTTCACTTACCAG<br>JVO-3214- AGTGTGGTAGCAGGATCG
Δ*steC*	JVS-3630	JVS-8711	JVO-3227- AGTTGCCAGATATCAGAAAGGTATTTAAGAGATATCATCGGTGTAGGCTGGAGCTGCTTC<br>JVO-3228- TTCATCCTTTAATACCTTAGCCACAAGAGTCCCTTCCTCCGGTCCATATGAATATCCTCCTTAG	JVO-3229- TCAGAGGATGAGACATATGC<br>JVO-3230- ATCTGTAGCGAATGTGCC
Δ*steA*	JVS-3632	JVS-8709	JVO-3219- AGGATATGCCATATACATCAGTTTCTACCTATGCCAGAGCGTGTAGGCTGGAGCTGCTTC<br>JVO-3220- TGAGGTAAGGCATTATATGCTGCCATTCGCTCTCTGACGGGGTCCATATGAATATCCTCCTTAG	JVO-3221- TCGTCATAATGAGAGAGGAGTAG<br>JVO-3222- AGAATCTCTTTGCGACACG
Δ*spiC*	JVS-3634	JVS-8691	JVO-3174- ATAGAAACTCCCATTTATGTCTGAGGAGGGATTCATGCGTGTAGGCTGGAGCTGCTTC<br>JVO-3175- TACTTGTGGTATAATAACCGTTTAACCATCCCCCATCCGCGGTCCATATGAATATCCTCCTTAG	JVO-3176- AGGTATATGTAGACAGCATCC<br>JVO-3177- AATAGCAATAAGCTCAGAGC
Δ*sseK2*	JVS-3637	JVS-8704	JVO-3182- TCCCGGATTAGAGGTTTGATTTCATGTCAAAGTAATACTCGTGTAGGCTGGAGCTGCTTC<br>JVO-3183- AATGCTGGATGCTCGCTGCGGTTAACAGCAATAATCCGGTCCATATGAATATCCTCCTTAG	JVO-3184- TTAGCATTGTGACGTTAACG<br>JVO-3185- AACCCTTTACTGAGCCAGTC
Δ*sspH2*	JVS-3639	JVS-8692	JVO-3215- TCAGTAATCGCCGCATTTATCGTATTGCCTGGTCTGATACGTGTAGGCTGGAGCTGCTTC<br>JVO-3216- TATCTTTGTCGCACCGCACCTCATTCACCTGGTGCATCAGGGTCCATATGAATATCCTCCTTAG	JVO-3217- ATGCCCTTTCATATTGGAAG<br>JVO-3218- ACCTGTACATCATCCGTACTG
Δ*steB*	JVS-3641	JVS-8710	JVO-3223- TCAGGTAATAATCCATGCCTATTTCGATTTGTAAACATGGGTGTAGGCTGGAGCTGCTTC<br>JVO-3224 - AGTGACAGGTTAGCAGATGTTTCAGGCCAAACCCAAGTTGGGTCCATATGAATATCCTCCTTAG	JVO-3225- TTCCAGGCTTAGTCAATGTG<br>JVO-3226- AAGGACATGGCATGACAC
Δ*sseJ*	JVS-3642	JVS-8702	JVO-3207- AGCAGTCAGATAATATGTACCAGGCATTAACCTCACGTTGGTGTAGGCTGGAGCTGCTTC<br>JVO-3208- ATGGACTTCCTGGGTTGGATGGACAAGGTCGTTGAAGACGGTCCATATGAATATCCTCCTTAG	JVO-3209- AGCTCATACTCACGCCAG<br>JVO-3210- ATCGGCAGCAAAGATAGC
Δ*sspH1*	JVS-3667	JVS-8698	JVO-3245- TCTGTAAGTATGCAGGCTATTGCTGGTGCAGCGGCACCAGGTGTAGGCTGGAGCTGCTTC<br>JVO-3246- AAGCGTTCCGGCACTTTGCGTTCCAGCACGCTGTGTAACGGGTCCATATGAATATCCTCCTTAG	JVO-3247- ATGTTTAATATCCGCAATACAC<br>JVO-3248- TCAGCACTTCGTCATACAG
Δ*sopD2*	JVS-4373	JVS-8697	JVO-4032- ATGTGTCCAAAGAACGTTATGCGCTGTCCAGCGCCTGGCGGTGTAGGCTGGAGCTGCTTC<br>JVO-4033- GAACAGCAGCAGGACGTTATTACTCGTACCTTCACGCAGACCATATGAATATCCTCCTTAG	JVO-3572-<br>GTTTTTTGACGTCCATAGCGCCCTGGTTTTACT<br>JVO-3037- GTTTTAACTCCCTGACGAAAG
Δ*ssaM*		JVS-8712	JVO-5227- AATATTCAGCTTTTTATTCAATTAGCAGGATTAGCTGAACGGTGTAGGCTGGAGCTGCTTC<br>JVO-5228- GAGCTCGGCGGAGCTGGAAAGAGGTGGAGAACGGTCCATATGAATATCCTCCTTAG	JVO-5229- GATTGGGATCTCATTACTGAAC<br>JVO-5230- GCGACTCCAGAAATTTTATTTG
Δ*ssaL*		JVS-8713	JVO-5231- GTTATTTCGCCTTCAATGTTAGCTCTCCAGGAGTTACGTGTAGGCTGGAGCTGCTTC<br>JVO-5232- TCAGAATAAAACCTGATTTATCTTTACTTCACGAAGCGTTTCGAGGGTCCATATGAATATCCTCCTTAG	JVO-5233- GCCAGGTTATAGAGGAACAAG<br>JVO-5234- GAGCCAGAAAGCCAATTTA
ΔSPI-2	JVS-1103	JVS-5294	The SPI-2 knockout strain was kindly provided by Karsten Tedin	
Δ*spiC*+pLuc		JVS-8714	JVS-8691 was transformed with the plasmid pXG0-amp	
Δ*spiC*+p*spiC*		JVS-8715	JVS-8691 was transformed with the plasmid pXG0-amp-SpiC	

Δ*spvB*+pLuc		JVS-8716	JVS-8707 was transformed with the plasmid pXG0-amp	
Δ*spvB*+p*spvB*		JVS-8717	JVS-8691 was transformed with the plasmid pXG0-amp-SpvB	
Δ*spvB*+ p*spvB_mut*		JVS-8718	JVS-8691 was transformed with the plasmid pXG0-amp-SpvB_mut	
