# Supplementary material for: A Candidate Approach Implicates the Secreted Salmonella Effector Protein SpvB in P-Body Disassembly
Source: PLoS One. 2011 Mar 1;6(3):e17296. doi: 10.1371/journal.pone.0017296 (PMC3046968; doi:10.1371/journal.pone.0017296)
Supplement: Table S2 — List of the primers used for cloning in pXG0-amp. (PDF) [file pone.0017296.s006.pdf]

Table S2. List of the primers used for cloning in pXG0-amp.

construct	Oligos
pXG0-amp-SpiC	JVO-5225- GTTTTTCTCGAGCAAATTGTAAGTTTTTATGTCAATGCTG<br>JVO-5226- GTTTTTTCTAGATTATACCCCACCCGAATAAAGTTTATG
pXG0-amp-SpvB	JVO-5848- GTTTTTCTCGAGGCCAGCCGGCTGTCACC<br>JVO-5849- GTTTTTTCTAGACAGCTACAATATTCAAAGGAGGGATG
pXG0-amp-SpvB_mut	JVO-5832- GTTGCACATTTTAAAGGAGACGCAGACATGCTTTTCCCTCC<br>JVO-5833- GGAGGGAAAAGCATGTCTGCGTCTCCTTTAAAATGTGCAAC
